# Beyond forest plots: trial sequential analysis of magnesium sulfate as an adjuvant in supraclavicular brachial plexus block

**DOI:** 10.1016/j.bjane.2026.844835

**Published:** 2026-09-20

**Authors:** Willyam Barros Saraiva, Rafael Wanderley Persiano Malta, Roberta Ribeiro Brandão Caldas, Olavo Barbosa de Oliveira-Neto, Fabiano Timbó Barbosa, Célio Fernando de Sousa-Rodrigues

**Affiliations:** a Universidade Federal de Alagoas, Hospital Universitário Professor Alberto Antunes, Maceió, AL, Brazil; b Sociedade Brasileira de Anestesiologia, Higher Degree in Anesthesiology (TSA), Rio de Janeiro, RJ, Brazil; c Universidade Federal de Alagoas, Maceió, AL, Brazil

Dear Editor,

Peripheral nerve block adjuvants are frequently investigated in regional anesthesia to prolong sensory and motor block duration and improve postoperative analgesia, particularly when the duration of local anesthetics alone may be insufficient. In this context, magnesium sulfate has been evaluated as a potential adjuvant to local anesthetics.^1^ Although conventional meta-analyses^2^ have suggested clinically relevant benefits, these conclusions are commonly based on cumulative forest plots, which are vulnerable to random error and inflation of type I error, especially when the number of trials is limited or when repeated significance testing is performed.

Trial Sequential Analysis (TSA) extends conventional meta-analysis by applying principles analogous to interim monitoring in randomized trials. TSA adjusts significance thresholds according to the accrued information size and constructs sequential monitoring boundaries, thereby distinguishing robust evidence from potentially spurious findings.^3^ Therefore, applying TSA to previously reported pooled estimates may help clarify which conclusions are statistically robust after adjustment for random error and which should remain hypothesis-generating.

## Methods

This TSA was based on the original meta-analytic database used in the previously published BJAN meta-analysis on magnesium sulfate as an adjuvant in supraclavicular brachial plexus block.^2^ No new literature search, study selection, or data extraction from primary randomized trials was performed. Outcome-level data, including effect estimates and corresponding measures of variance, were retrieved from the original meta-analytic dataset and checked for consistency with the pooled estimates reported in the published meta-analysis. This approach was chosen because the objective of the present study was to reassess whether the original pooled estimates crossed prespecified TSA monitoring boundaries after adjustment for random error and accrued information size. Thus, the TSA was designed as a methodological reassessment of the previously reported meta-analytic estimates rather than as a new effectiveness analysis based on independently defined clinical thresholds. The original meta-analysis included randomized controlled trials comparing local anesthetics with or without magnesium sulfate as an adjuvant for supraclavicular brachial plexus block in adult patients. Additionally, the authors followed a structured checklist^4^ based on the EQUATOR Network Reporting Guidelines (https://www.equator-network.org/reporting-guidelines/).

The post-publication status of the randomized trials included in the original dataset and all references cited in this letter to the editor was checked using the Retraction Watch Database, PubMed/MEDLINE, and publishers’ websites, including CrossMark when available. No retraction notices or expression of concern were identified.

The following outcomes were evaluated: onset of sensory block, onset of motor block, duration of sensory block, duration of motor block, and rescue analgesic consumption within 24 hours.

TSA was conducted using TSA software.^5^^,^^6^ For each continuous outcome, the Required Information Size (RIS) was calculated using a random-effects model. The type I error risk was set at 5% using a two-sided alpha, and the type II error risk was set at 20%, corresponding to 80% statistical power. The anticipated intervention effect was defined according to the pooled effect estimate obtained in the previously published conventional meta-analysis. Between-study heterogeneity was incorporated into the RIS using the diversity/heterogeneity adjustment provided by the TSA software. Sequential monitoring boundaries were constructed using the O’Brien-Fleming approach, because this method applies conservative thresholds during early cumulative evidence accrual and reduces the risk of false-positive conclusions due to repeated significance testing. A cumulative *Z*-curve crossing the trial sequential monitoring boundary for benefit was interpreted as evidence that the previously reported pooled estimate was robust after adjustment for random error and accrued information size. Failure to cross the monitoring boundary was interpreted as inconclusive sequential evidence.

## Results

Outcome-specific TSA assumptions are presented in Supplementary Table 1.

### Block duration

For sensory block duration, 10 studies contributing 11 comparison units and 734 participants were included in the final TSA. The Diversity-Adjusted Required Information Size (DARIS) was reached, and the final cumulative Z-curve crossed the O’Brien-Fleming monitoring boundary, favoring magnesium sulfate (Fig. 1A).

**Figure 1:**
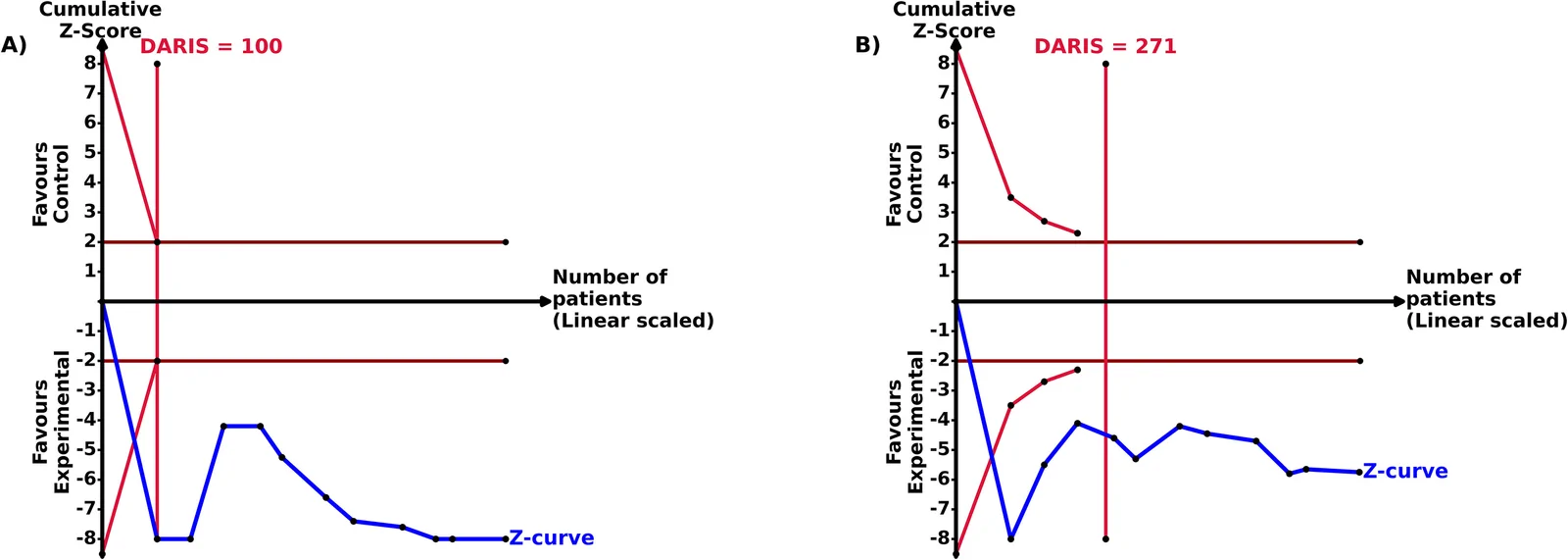
TSA for duration outcomes: (A) Sensory block duration and (B) Motor block duration. The x-axis shows the cumulative information size, and the y-axis shows the cumulative *Z*-score. Blue lines represent cumulative *Z*-curves; red lines represent conventional significance boundaries and O’Brien-Fleming trial sequential monitoring boundaries; the vertical red line indicates the Diversity-Adjusted Required Information Size (DARIS). Favours experimental: local anesthetic plus magnesium sulfate; and favours control: local anesthetic alone.

For motor block duration, 10 studies contributing 11 comparison units and 734 participants were included in the final TSA. The DARIS was reached, and the final cumulative *Z*-curve crossed the O’Brien-Fleming monitoring boundary, favoring magnesium sulfate (Fig. 1B).

### Block onset

For sensory block onset, 10 studies contributing 11 comparison units and 734 participants were included in the final TSA. The DARIS was reached; however, the final cumulative *Z*-curve did not clearly cross the O’Brien-Fleming monitoring boundary, although it crossed the conventional significance boundary (Fig. 2A). Therefore, this outcome was considered inconclusive after sequential adjustment.

**Figure 2:**
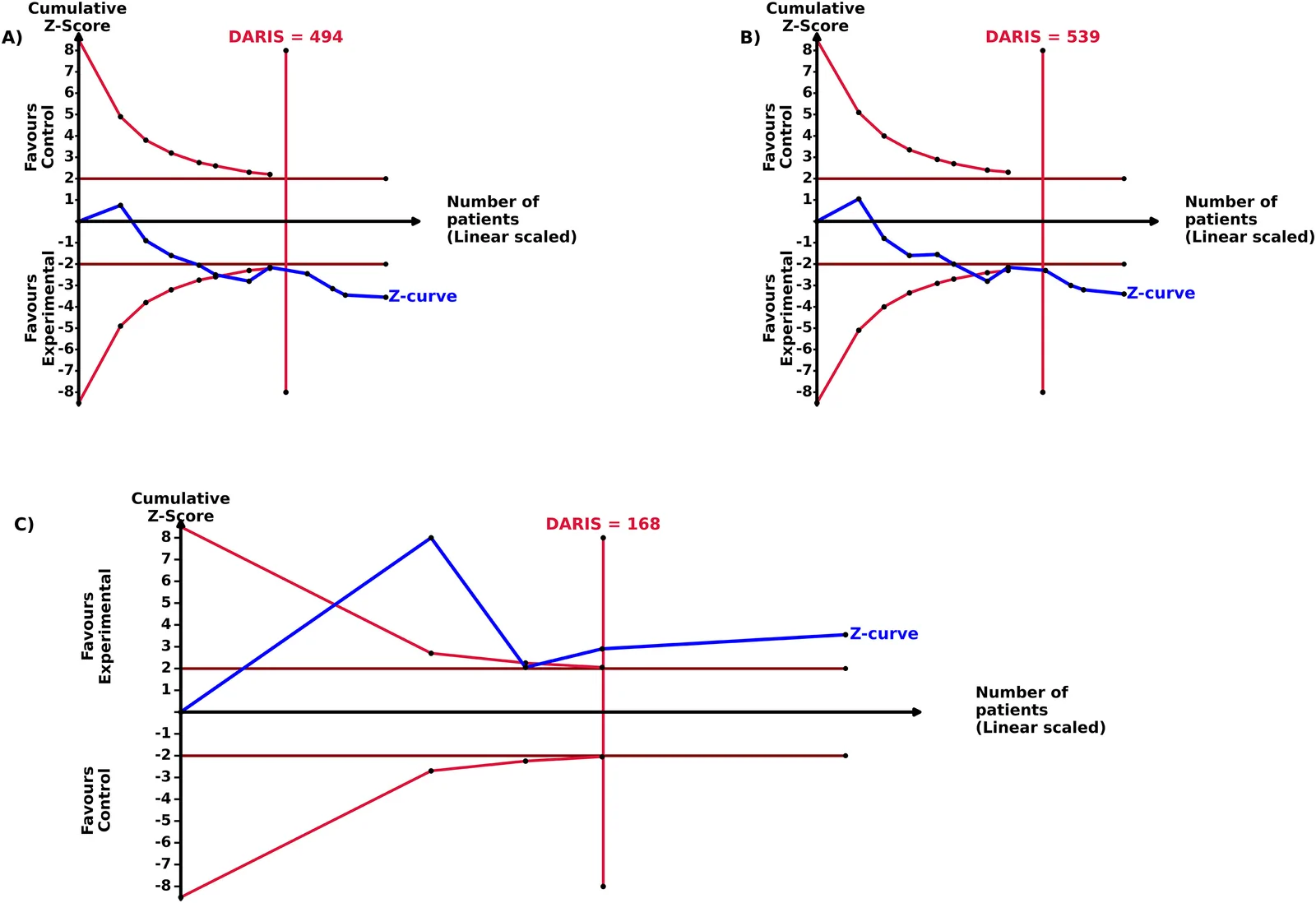
TSA for onset and postoperative analgesia outcomes: (A) Sensory block onset, (B) Motor block onset, and (C) Rescue analgesic consumption within 24 hours. The x-axis shows the cumulative information size, and the y-axis shows the cumulative *Z*-score. Blue lines represent cumulative *Z*-curves; red lines represent conventional significance boundaries and O’Brien-Fleming trial sequential monitoring boundaries; the vertical red line indicates the Diversity-Adjusted Required Information Size (DARIS). Favours experimental: local anesthetic plus magnesium sulfate; and favours control: local anesthetic alone.

For motor block onset, 10 studies contributing 11 comparison units and 734 participants were included in the final TSA. The DARIS was reached; however, the final cumulative *Z*-curve did not clearly cross the O’Brien-Fleming monitoring boundary, despite crossing the conventional significance boundary (Fig. 2B). Therefore, this outcome was also considered inconclusive after sequential adjustment.

### 24-hour rescue analgesic consumption

For 24-hour rescue analgesic consumption, 4 studies contributing 4 comparison units and 266 participants were included in the final TSA. The DARIS was reached, and the final cumulative *Z*-curve crossed the O’Brien-Fleming monitoring boundary, favoring magnesium sulfate (Fig. 2C).

## Discussion

The present TSA provides a methodological reassessment of previously published pooled estimates on magnesium sulfate as an adjuvant in supraclavicular brachial plexus block, rather than new clinical evidence regarding the intervention itself.

Sequential monitoring is particularly relevant in anesthesiology research, where meta-analyses often rely on a limited number of relatively small, randomized trials. In such settings, repeated significance testing increases the risk of type I error, potentially leading to premature or spurious conclusions. TSA addresses this limitation by applying adjusted significance thresholds and by estimating the DARIS, thereby offering a framework analogous to interim analyses in single randomized controlled trials. Our findings illustrate this principle: for sensory and motor block duration, the cumulative *Z*-curves crossed the prespecified TSA monitoring boundaries under the stated assumptions, whereas outcomes related to block onset did not cross these boundaries despite nominal statistical significance in conventional analyses.

For 24-hour rescue analgesic consumption, the TSA indicated that the cumulative information size exceeded the DARIS, and the final cumulative Z-curve crossed the prespecified O’Brien-Fleming monitoring boundary, favoring magnesium sulfate under the stated assumptions. This suggests that, within the TSA framework, the observed reduction in rescue analgesic consumption was unlikely to be explained by random error alone. However, TSA addresses the risk of random errors due to sparse and repeated testing; it does not by itself resolve other sources of uncertainty, such as clinical heterogeneity, risk of bias, or outcome-level differences across trials.

Taken together, these results emphasize that TSA should be viewed not as a replacement for conventional meta-analysis, but as a complementary tool that distinguishes robust evidence from findings that remain hypothesis-generating. For magnesium sulfate as an adjuvant in supraclavicular block, TSA suggests that the previously reported prolongation of block duration and reduction in 24-hour rescue analgesic consumption are statistically robust, whereas the pooled estimates for block onset remain uncertain and should be interpreted as hypothesis-generating.

## Conclusion

This TSA supports the robustness against random error of the previously reported benefits of magnesium sulfate on sensory and motor block duration and 24-hour rescue analgesic consumption in supraclavicular brachial plexus block. For these outcomes, the cumulative information size exceeded the DARIS and the cumulative *Z*-curves crossed the prespecified O’Brien-Fleming monitoring boundaries under the stated TSA assumptions. In contrast, sensory and motor block onset did not clearly cross the monitoring boundaries and therefore remained inconclusive after sequential adjustment. These findings clarify which pooled estimates are statistically robust within the TSA framework and which remain uncertain after adjustment for random error.

These findings should not be interpreted as a recommendation for routine perineural magnesium sulfate use, which remains off-label. Interpretation should remain cautious given trial heterogeneity, the secondary nature of this methodological reanalysis, and certainty limitations in the original evidence base. Further adequately powered randomized trials with standardized dosing, outcome reporting, and safety assessment are still needed.

## Data availability statement

The complete dataset and materials supporting this Trial Sequential Analysis have been deposited and are publicly available at https://doi.org/10.5281/zenodo.20891978. The deposited files include the original study-level extraction dataset used in the prior meta-analysis, TSA input files/settings, and outputs. No statistical code was used for TSA; analyses were performed using TSA software.

## AI assistance disclosure

The authors used AI-assisted technology (ChatGPT, OpenAI) exclusively for linguistic support, including grammar revision and translation. All content was critically reviewed and approved by the authors.

## Funding

The authors declare that no financial support or funding was received for this study.

## Conflicts of interest

The authors declare no conflicts of interest.

## Appendix Supplementary materials

Image, application 1

## Footnotes

Supplementary material associated with this article can be found in the online version at doi:10.1016/j.bjane.2026.844835.

## References

[1] Li M., Jin S., Zhao X. (2016). Does magnesium sulfate as an adjuvant of local anesthetics facilitate better effect of perineural nerve blocks? A meta-analysis of randomized controlled trials. Clin J Pain.

[2] Saraiva W.B., Candido I.E., Caldas R.R.B., Barbosa FT. (2026). Efficacy of magnesium sulfate as an adjuvant to local anesthetics in supraclavicular brachial plexus block: a meta-analysis of randomized trials. Braz J Anesthesiol.

[3] Kang H. (2021). Trial sequential analysis: novel approach for meta-analysis. Anesth Pain Med (Seoul).

[4] Coskun Benlidayi I., Gupta L. (2024). Letter to the Editor Standards (LETTERS): considerations for authors, reviewers, and editors. J Korean Med Sci.

[5] Thorlund K., Engstrøm J., Wetterslev J., Brok J., Imberger G., Gluud C. (2011).

[6] Claire R., Gluud C., Berlin I., Coleman T., Leonardi-Bee J. (2020). Using trial sequential analysis for estimating the sample sizes of further trials: example using smoking cessation intervention. BMC Med Res Methodol.

