# Supplementary material for: Beyond forest plots: trial sequential analysis of magnesium sulfate as an adjuvant in supraclavicular brachial plexus block
Source: Braz J Anesthesiol. 2026 Sep 20;76(6):844835. doi: 10.1016/j.bjane.2026.844835 (PMC13651671; doi:10.1016/j.bjane.2026.844835)
Supplement: Supplementary file 1 [file mmc1.docx]

BJAN-D-26-00197_Supplementary Material

**Supplementary Table 1** Trial sequential analysis assumptions and monitoring-boundary status for magnesium sulfate outcomes.

|  | **Number of studies** | **Comparison units** | **Participants** | **Anticipated effect** | **Diversity adjustment** | **Variance used for DARIS calculation** | **DARIS** | **Final Z-curve status relative to the monitoring boundary** |
| --- | --- | --- | --- | --- | --- | --- | --- | --- |
| **Onset of Sensory Block** | 10 | 11 | 734 | MD = 3.9132 min | 99.19% | 1.7564 | 494 | Did not clearly cross O’Brien-Fleming monitoring boundary; crossed conventional significance boundary after DARIS was exceeded. |
| **Onset of Motor Block** | 10 | 11 | 734 | MD = 4.7259 min | 99.26% | 2.6434 | 539 | Cumulative *Z*-curve crossed the conventional significance boundary after the DARIS was exceeded; it did not clearly cross the O’Brien-Fleming boundary. |
| **Duration of Sensory Block** | 10 | 11 | 734 | MD = 180.8373 min | 99.0% | 340.5815 | 100 | Cumulative *Z*-curve crossed the O’Brien-Fleming monitoring boundary, favoring magnesium sulfate. |
| **Duration of Motor Block** | 10 | 11 | 734 | MD = 151.2556 min | 99.26% | 931.9726 | 271 | Cumulative *Z*-curve crossed the lower O’Brien-Fleming monitoring boundary, favoring magnesium sulfate. |
| **24-hour Rescue Analgesic Consumption** | 4 | 4 | 266 | MD = -27.9175 | 86.98% | 545.3479 | 168 | Cumulative Z-curve crossed the O’Brien-Fleming monitoring boundary after the DARIS was exceeded, favoring magnesium sulfate. |
